# The effcet of annulus fibrosus suture combined with percutaneous transforaminal endoscopic discectomy on obese patients with lumbar disc herniation

**DOI:** 10.3389/fpain.2025.1568227

**Published:** 2025-03-14

**Authors:** Leyu Zhao, Qi Yan, Lijie Yuan, Tianyi Wu, Yun Teng, Junjie Niu, Dawei Song, Jinning Wang, Xiao Sun, Rui Chen, Xianggu Zhong, Jiarong Li, Xiaolan Gu, Jun Zou

**Affiliations:** ^1^ Department of Orthopedics, The First Affiliated Hospital of Soochow University, Suzhou, China; ^2^ Department of Orthopaedic Surgery, Taicang Affiliated Hospital of Soochow University, Taicang, Jiangsu, China; ^3^ Department of Orthopaedic Surgery, Wuzhong People’s Hospital, Suzhou, Jiangsu, China

**Keywords:** annulus fibrosus suture, percutaneous transforaminal endoscopic discectomy, obesity, lumbar radiculopathy, lumbar disc herniation

## Abstract

**Background:**

Lumbar disc herniation (LDH) is a prevalent degenerative disc disorder frequently resulting in lumbar and leg pain. Obese patients with LDH often encounter the scenario where the disc herniation is not completely resolved in the short term following percutaneous transforaminal endoscopic discectomy (PTED), necessitating subsequent surgical intervention, or where long-term reherniation occurs post-procedure. Currently, the literature provides little information regarding the application of annulus fibrosus suture (AFS) as a supplementary measure to PTED for diminishing the recurrence of disc herniation. Our aim was to evaluate the short-term and long-term therapeutic outcomes of combining PTED with AFS, with a particular focus on the impact of AFS on the recurrence rate of disc herniation following PTED.

**Methods:**

We recruited 23 obese patients with single-level LDH diagnosed between December 2021 and December 2023. All patients successfully underwent PTED in conjunction with AFS and the postoperative follow-up. We collected and analyzed data related to baseline parameters, disc degeneration grading, clinical effectiveness, surgery-related factors, lumbar spine function, pain severity, quality of life, and adverse prognosis events.

**Results:**

Compared with preoperative assessments, all patients exhibited significant improvements in Visual Analog Scale for leg pain (VAS-LP), Oswestry Disability Index (ODI), and Japanese Orthopaedic Association (JOA) scores (*P* < 0.05). During the short-term follow-up period, no patient required a secondary conventional microdiscectomy due to severe complications. At the one-year follow-up, no patient experienced significant recurrent radicular leg pain that would raise suspicion of LDH recurrence. However, when PTED was combined with AFS, the improvement in Visual Analog Scale for back pain (VAS-BP) was relatively less pronounced.

**Conclusions:**

The synergy of PTED and AFS seems to be a comparatively safe and efficacious approach for treating LDH in obese patients. AFS reduces the incidence of long-term recurrent leg pain, which may in turn reduce the probability of LDH recurrence after PTED. Consequently, AFS should be regarded as an efficacious supplementary procedure to PTED, adept at efficiently reducing the recurrence rate in obese individuals with LDH.

## 1 Introduction

The severity of lumbar radiculopathy due to lumbar disc heriation (LDH), primarily characterized by leg pain, significantly impacts patients' daily activities and overall quality of life, often serving as a critical factor in determining the need for surgical intervention (1–4). In patients suffering from persistent sciatica, early discectomy demonstrates superior effectiveness in relieving radicular leg pain compared to conservative treatment (5–7). In comparison to nonoperative treatment modalities, lumbar discectomy has been demonstrated to elicit more robust therapeutic effects with regard to the amelioration of lumbar radiculopathy (8). However, given an average postoperative follow-up period exceeding one year, the incidence of recurrent lumbar disc herniation (rLDH) subsequent to conventional lumbar discectomy remains between 5% and 10%, and can still reach up to 10% in recent years, resulting in enduring postoperative recurrent radicular leg pain and substantially augmenting healthcare expenditures (4, 9–13). percutaneous transforaminal endoscopic discectomy (PTED) is an emerging minimally invasive technique in spinal surgery that demonstrates comparable therapeutic efficacy and safety to conventional lumbar discectomy for treating LDH, offering evident minimally invasive advantages and promoting rapid postoperative recovery (14, 15). However, it fails to demonstrate a statistical advantage in relation to recurrence rate of rLDH and the incidence of postoperative recurrent radicular pain (16, 17). Thus, there is an urgent need in clinical practice for a new approach to diminish the recurrence rate following intervertebral disc surgery. Obesity is frequently considered a risk factor for LDH due to its detrimental impact on spinal biomechanics (18, 19). Moreover, the recurrence rate after discectomy in obese patients can even reach 10% to 15% (20, 21). Therefore, in comparison to non-obese patients, the long-term radicular leg pain linked to postoperative recurrence is a more critical concern for obese patients who have undergone discectomy.

It is currently asserted that annular defects correlate with symptom recurrence and the risk of reoperation, whereas annular competence can predict the recurrence rate following lumbar discectomy for leg pain (22–24). Annular defects not only furnish an anatomical aperture for rLDH but also engender conditions conducive to inflammation-induced neuropathic pain (24–27). Against this backdrop, the urgent development of a suture technique for the torn annulus fibrosus post-lumbar discectomy is essential. In the initial phases of study, the extensive surgical incisions and disruption of the neural environment associated with conventional lumbar discectomy led to considerable postoperative adhesions. Additionally, the lack of devices designed to simplify the suturing process increased the cost of repairing the annulus fibrosus (28, 29). These issues cumulatively led to unsatisfactory therapy outcomes and engendered pessimism about the achievement of the expected benefits of annulus fibrosus repair. Hence, the reparation of annular defects persists in presenting challenges. PTED, characterized by its minimal invasiveness and negligible harm to surrounding anatomical structures, is a novel approach for annular repair. Concurrently, the evolution of the Disposable Annulus Stapler has kindled a glimmer of hope for the therapeutic use of Annulus Fibrosus Suture (AFS). preliminary trials are commencing to assess the therapeutic effectiveness of employing a disposable annulus stapler for the treatment of annular defects subsequent to PTED (30). However, there is seldom literature reporting the effects of AFS on reducing the recurrence rate and alleviating residual pain after PTED. Moreover, a study gap persists about the therapeutic outcomes in obese patients following the synergy of PTED and AFS. To verify our hypothesis that the combination of AFS and PTED may effectively reduce the recurrence rate and alleviate residual pain after PTED in obese patients, we conducted a retrospective study, aiming to evaluate the potential for large-scale clinical application of this combined approach in obese patients with LDH.

## 2 Materials and methods

### 2.1 Patients

This retrospective study included 23 obese patients diagnosed with single-level LDH from December 2021 to December 2023. All patients successfully underwent PTED + AFS surgery. All participants received a comprehensive explanation of the benefits and risks associated with AFS and made informed decisions regarding their surgical intervention. All procedures involving human participants were conducted in accordance with the Declaration of Helsinki. The Institutional Review Board waived the necessity for informed consent because of the retrospective study design.

#### 2.1.1 Inclusion criteria

The criteria for inclusion were as follows: (1) BMI ≥ 30 kg/m^2^; (2) Clinically diagnosed with single-level lumbar disc herniation; (3) Symptoms including chronic low back pain with radiating pain in the extremities, limited lumbar mobility, and a positive straight leg raise test; (4) Clinical diagnosis of lumbar disc herniation with consistent clinical symptoms, signs, and imaging findings; (5) Poor response or recurrent episodes after 12 weeks of conservative treatment; (6) Complete clinical data before surgery and follow-up records for more than 1 years post-surgery; (7) Patient consent for annular suturing, performed after discectomy with a relatively intact and defect-free annulus fibrosus.

#### 2.1.2 Exclusion criteria

The criteria for exclusion were as follows: (1) Patients with multilevel LDH; (2) Presence of lumbar spondylolisthesis, spinal stenosis, spinal infection, bone tuberculosis, lumbar tumors, or other lumbar diseases; (3) Clinical symptoms and signs inconsistent with imaging findings; (4) History of previous lumbar spine surgery; (5) Severe osteoporosis and significant calcification of the affected segment; (6) Annulus fibrosus rupture unsuitable for suturing; (7) Cognitive or mental disorders.

### 2.2 Surgical procedure

**Figure 1:**
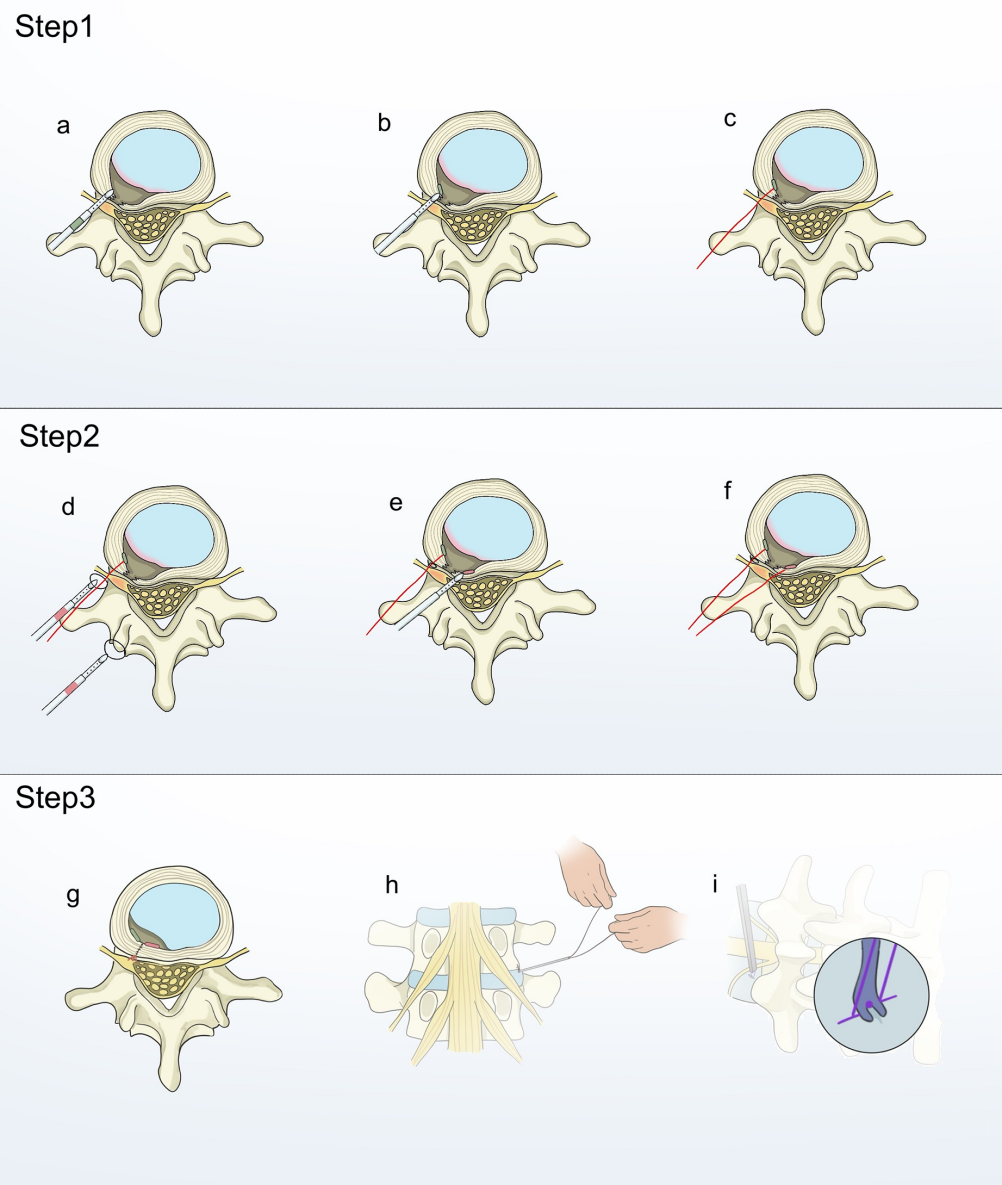
Step 1: Schematic diagram of annulus defect closure using a double-stitch suture technique. The disposable annulus stapler was inserted into the annulus on one side of the defect **(a)** The slider at the tail end of the gun-type suturing device was pushed forward to perform transverse anchoring **(b)** The gun-type annulus fibrosus suturing device was withdrawn, and the suture was pulled upward to confirm the security of the first stitch **(c)** Step 2: The suture loop of the second stitch was passed over the suture of the first stitch **(d)** The second stitch performed transverse anchoring **(e,f)**. Step 3:The suture was tightened to secure the first knot **(g)** Form the second knot externally and use a minimally invasive endoscopic knot pusher to advance it towards the first knot **(h,i)**.

Patients were positioned prone under local anesthesia. The surgeon performed routine disinfection and draping. A guide needle was put in with the help of C-arm fluoroscopy, and a working cannula was connected to the foraminoscopic system. Under direct visualization with the foraminoscope, the protruding nucleus pulposus was identified. The annulus fibrosus was either incised or the protruding portion was excised from an existing rupture in the annulus fibrosus, with strict protection of the annular fissure during the procedure. An EFit Disposable Annulus Stapler was required for this study. Subsequently, the Disposable Annulus Stapler was inserted into the annulus fibrosus on one side of the defect, taking care to sense the toughness of the annulus and the depth of penetration. Once a sufficient depth was reached, the slider on the tail end of the gun-shaped suturing device was pushed forward until it could no longer be moved. At this point, the transverse anchor device at the head end of the gun-shaped suturing device would gradually emerge from the head end to the deep side of the annulus (Figure 1a). The transverse anchor device would rotate 90 degrees on its own and lock under the annulus fibrosus once it was fully out of the head end of the gun-shaped suturing device (Figure 1b). The gun-shaped annular suturing device was then removed, and the suture was pulled upward to confirm the security of the first stitch (Figure 1c). Another annular suturing device was used to puncture the other side of the defect. The head end of the second annular suturing device would have a suture loop. Before performing the second puncture, the suture of the first stitch was threaded through this loop (Figure 1d). The procedure for the second puncture mirrored that of the initial one, except that the deployment of the transverse anchor device was facilitated by a push rod (Figures 1e,f). Since the suture from the first stitch had been previously passed through the suture loop of the second stitch, tightening the suture was sufficient to secure the first knot (Figure 1g). Subsequently, a second knot was formed outside the body, and an endoscopic knot pusher, specifically designed for minimally invasive procedures, was employed to advance the second knot toward the first, ensuring a robust ligation (Figures 1h,i). After irrigating the incision with 0.9% sodium chloride solution and achieving hemostasis with radiofrequency, the working channel was removed, followed by routine suturing and dressing of the incision. Postoperatively, patients routinely received mecobalamin, mannitol, and other anti-inflammatory, dehydrating, and analgesic treatments. On the first postoperative day, all patients were guided to perform in-bed functional exercises, including straight-leg raising exercises and five-point or three-point support exercises. Medical staff also assisted patients in attempting ambulation. Patients were informed that they could begin swallowtail (fly-like) functional exercises on the third postoperative day. To ensure the scientific rigor of postoperative rehabilitation training after discharge, all patients received educational instructions from our team prior to discharge. Surgeons with similar experience and skill recorded the surgical time, postoperative hospital stay, incision length, working channel placement time, and intraoperative blood loss for both groups.

### 2.3 Follow-up and observation indexes

This study used the Pfirrmann grading system to assess the degree of intervertebral disc degeneration in patients prior to surgery. We used the Modified MacNab Criteria to statistically assess the clinical outcomes at one year postoperatively. The ODI and JOA scores at one month, three months, six months, and one year after surgery served as indicators to evaluate the recovery of lumbar spine function. The VAS was utilized to assess postoperative low back pain (VAS-BP) and leg pain (VAS-LP). Special attention was paid to the incidence of severe short-term complications and the recurrence rate at six months postoperatively. A recurrence of lumbar disc herniation was suspected to have occurred if, compared to the condition at one month postoperatively, patients experienced aggravated low back or leg pain, or a decline in lumbar spine function.

### 2.4 Statistical analysis

The software utilized for statistical analysis was IBM SPSS Statistics 25.0 (IBM Corp, Armonk, NY, USA). Repeated measures analysis of variance (ANOVA) was conducted to compare the mean scores of VAS-LP, VAS-BP, JOA score, and ODI in patients from preoperatively to postoperatively over time. The significance threshold was rigorously set at a *p*-value of 0.05. Statistical graphs were created using GraphPad Prism version 10.0.

## 3 Results

### 3.1 Basic characteristics of patients

23 obese patients underwent PTED combined with AFS for the treatment of LDH. Clinical data were collected before and after the procedure. The study included 11 male patients (47.83%) and 12 female patients (52.17%), with ages ranging from 21 to 61 years. All patients were diagnosed with single-level LDH. Preoperatively, 9 patients (39.13%) were graded as Pfirrmann Grade III, and 14 patients (60.87%) were graded as Pfirrmann Grade IV (Tables 1,2).

**Table 1:** Patient gender, lumbar disc herniation segment, and pfirrmann grade.

Categorical data	Cases(n)	Percentage
Gender		
Male	11	47.83%
Female	12	52.17%
Segment		
L4/5	9	39.13%
L5/S1	14	60.87%
Pfirrmann Grade		
III	9	39.13%
IV	14	60.87%

**Table 2:** Patient height, weight, and body mass index.

Continuous data	Mean	Median	Standard deviation	Min	Max
Ages(years)	42.00	42	10.70	21	61
Height(cm)	167.26	170	7.39	154	178
Weight(kg)	91.09	92	9.05	77	115
BMI	32.51	32.11	1.67	30.99	39.79

### 3.2 Patient treatment-related factors

**Figure 2:**
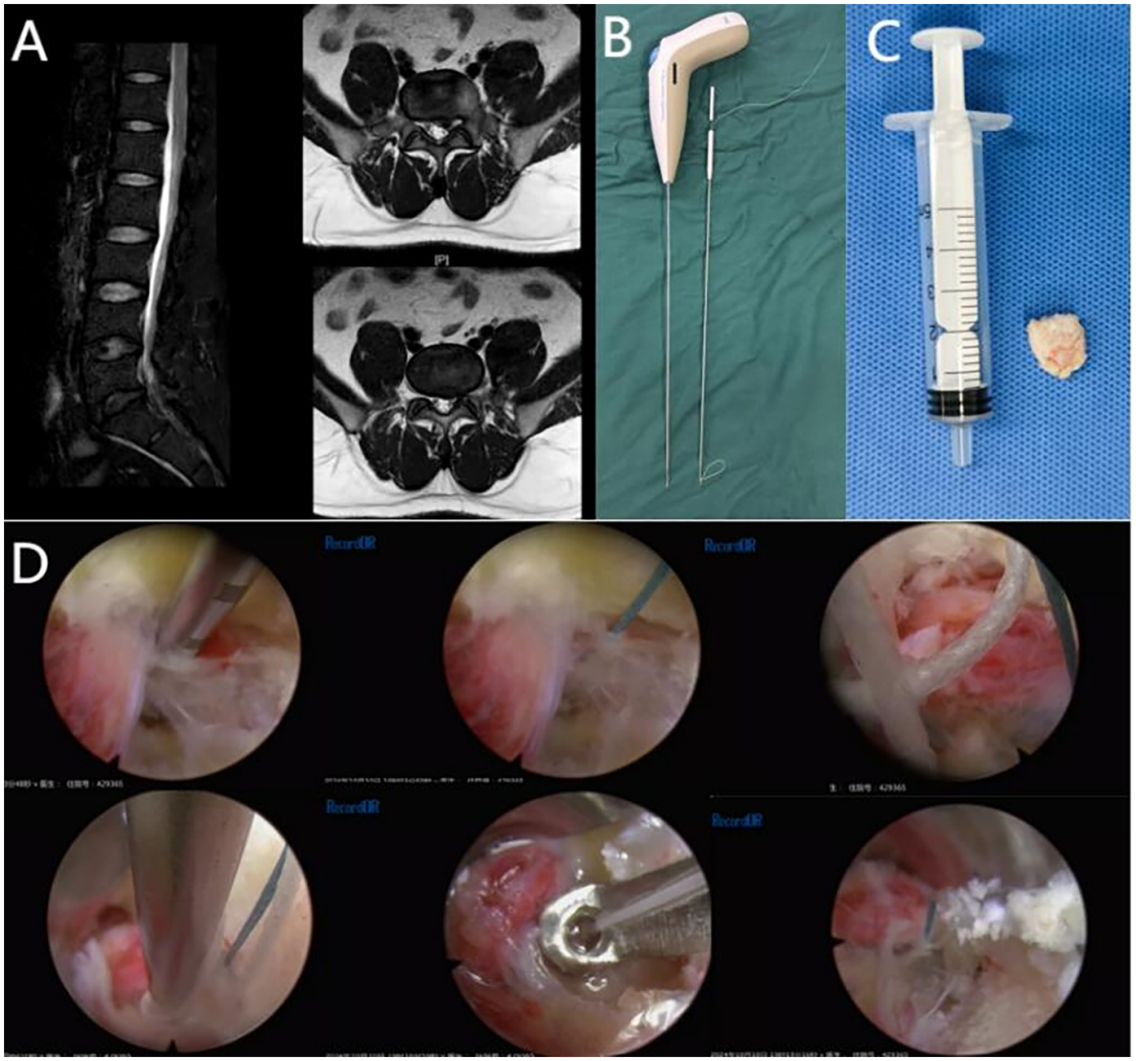
Preoperative MRI (L5-S1) and intraoperative data of the representative case are shown in the figures. **(A)** Preoperative MRI images of a 43-year-old obese patient with lumbar disc herniation (L5-S1). **(B)** The Disposable Annulus Stapler. **(C)** The nucleus pulposus removed from the patient during PTED. **(D)** Post-PTED double-stitch suture technique for annulus defect closure.

All patients successfully underwent PTED + AFS surgical intervention, with no significant surgical accidents occurring during the surgery (Table 3). Preoperative MRI (L5-S1) and intraoperative data of the representative case are shown in the figures (Figure 2).

**Table 3:** Intraoperative parameters of patients and postoperative hospital stay.

Treatment-related factors	Mean	Median	Standard sdeviation	Min	Max
Conservative treatment duration (weeks)	13.39	13	1.63	12	18
Course of disease duration (months)	10.43	8	5.31	5	23
Surgery time(min)	59.26	59.4	4.33	52.5	68.2
Incision length(cm)	0.77	0.71	0.17	0.6	1.19
Bleeding volume(ml)	47.81	47.5	6.24	37.4	58.1
Cannula placement time(min)	26.07	25.4	4.32	18.1	33.5
Postoperative hospital stay(day)	3.65	3	1.15	2	6

### 3.3 Patient clinical functional score

**Figure 3:**
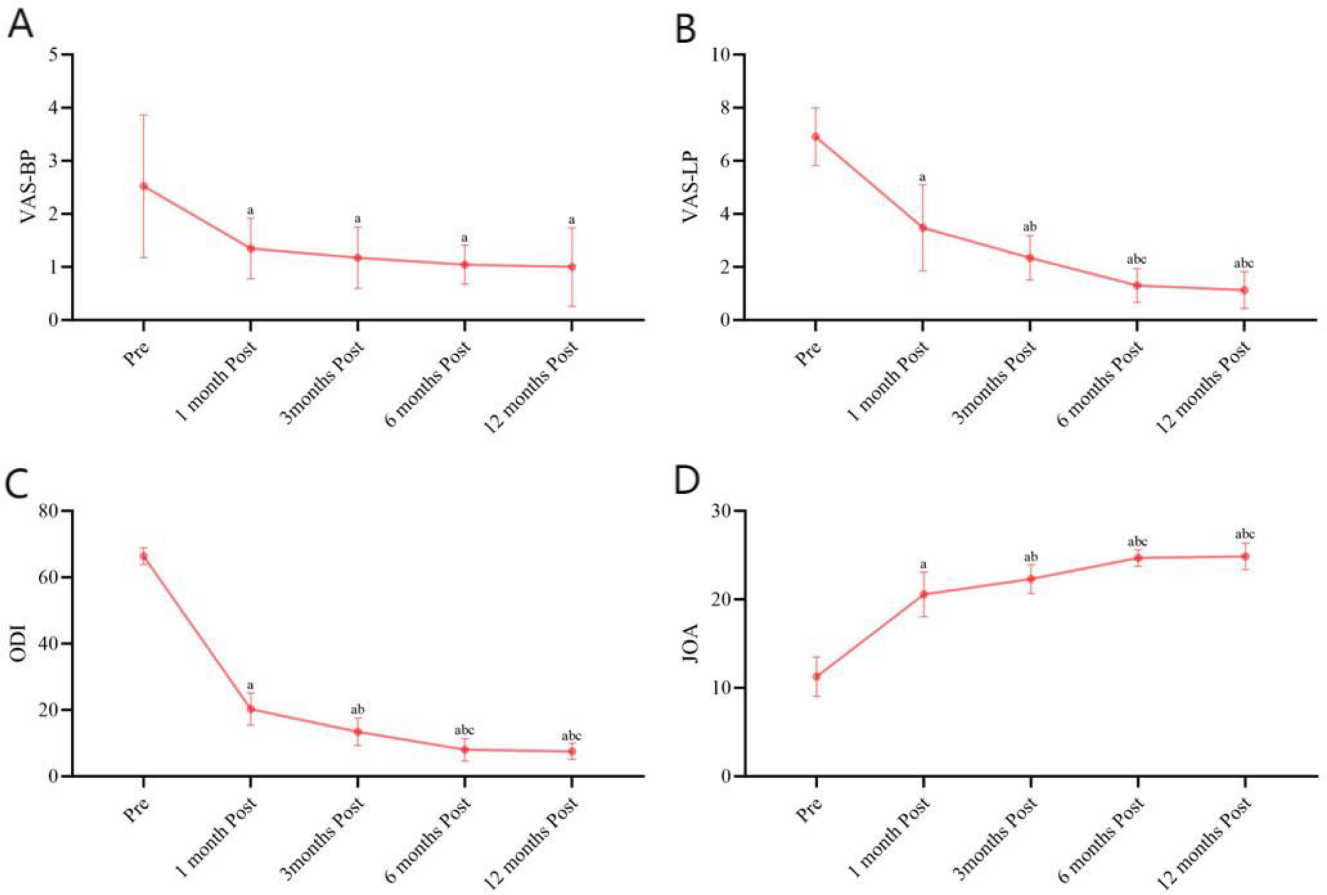
Preoperative and postoperative (1, 3, 6, and 12 months) VAS-LP, VAS-BP, ODI, and JOA scores. **(A)** Preoperative and postoperative (1, 3, 6, and 12 months) VAS-BP. **(B)** Preoperative and postoperative (1, 3, 6, and 12 months) VAS-LP. **(C)** Preoperative and postoperative (1, 3, 6, and 12 months) ODI. **(D)** Preoperative and postoperative (1, 3, 6, and 12 months) JOA scores.

Repeated-measures ANOVA was conducted on VAS-LP, VAS-BP, JOA, and ODI scores, and it showed that there were significant differences between time points (*P* < 0.05). Further LSD multiple comparisons indicated that scores at postoperative month 1 were lower than preoperative levels, scores at postoperative month 3 were lower than both preoperative and postoperative month 1 levels. There were no differences between postoperative months 6 and 12 in VAS-LP scores, although both were lower than preoperative, postoperative month 1, and postoperative month 3 levels (Figure 3A). B. For VAS-BP, there were no differences among postoperative months 1, 3, 6, and 12, but all were lower than preoperative levels (Figure 3B). Regarding ODI scores, there were no differences between postoperative months 6 and 12, but both were higher than preoperative, postoperative month 1, and postoperative month 3 levels. Additionally, scores at postoperative month 3 were higher than both preoperative and postoperative month 1 levels, with no difference between postoperative months 6 and 12, although both were higher than preoperative, postoperative month 1, and postoperative month 3 levels (Figure 3C). JOA scores were higher than preoperative levels at postoperative month 1, higher than both preoperative and postoperative month 1 levels at postoperative month 3, and higher than preoperative, postoperative month 1, and postoperative month 12 levels at postoperative month 6 (Figure 3D).

After one year of follow-up, the excellent and good rate of PTED combined with AFS can reach 91.30% (Table 4). It is worth mentioning that we observed no patients experiencing severe short-term complications requiring secondary surgical intervention, and no patients presented with severe recurrent radicular pain at the one-year follow-up.

**Table 4:** Macnab scores at one-year follow-up.

Level	Excellent	Good	Fair	Poor
Cases (n)	12	9	2	0

## 4 Discussion

Irrespective of the surgical approach—be it conventional lumbar discectomy, microsurgical lumbar discectomy, or the minimally invasive PTED—patients may still be at risk for recurrent LDH (rLDH) and the sequelae of persistent postoperative disability and pain, which can substantially impair their quality of life (31–33).The competence of the annulus is considered an important factor influencing the risk of rLDH after discectomy, and larger annular defects may be associated with accelerated disc height loss postoperatively (22, 34, 35). In fact, annular defects left after discectomy often heal poorly. Peripheral healing in injured discs may not occur, or if it does, it may only result in a thin layer of tissue (36). In the presence of a highly hydrated nucleus pulposus, the increased tensile stress on the inner annulus fibrosus associated with the damaged outer layer following discectomy initially leads to deformation and bulging of the collagen bundles, and ultimately results in the inner extension and persistent opening of the tear (37). This limited healing allows for the re-diffusion of nuclear irritants to surrounding neural structures under even slight increases in intradiscal pressure. When the annular defect fails to close effectively, the swelling pressure of the annulus fibrosus decreases with degeneration and injury. The loss of swelling pressure leads to increased matrix deformation, weakened strength and elasticity of the fiber structure, and the inability of the intervertebral disc (IVD) to effectively absorb and distribute compressive loads, thereby increasing the risk of rLDH (38). The integrity of the lamellar structure of the annulus fibrosus ensures its anisotropic and inhomogeneous characteristics. Annular defects may lead to weakened or disrupted interactions between fiber populations in the multilamellae annulus fibrosus, resulting in decreased tensile strength and stiffness, while increasing its deformability. Consequently, the annulus becomes more susceptible to deformation under mechanical overload, potentially further preventing the closure of the annular defect (39–41). Radial annular defects or posterior disc protrusions can lead to a decrease in the pressure of the adjacent disc nucleus, while simultaneously doubling the stress peaks within the annulus fibrosus. This accentuates the transfer of load from nucleus to annulus, further compromising the structural integrity of the annulus fibrosus (42). Thus, this creates a vicious cycle in which the presence of annular defects keeps making the loss of annular competence worse, which is bad for patients' long-term outcomes after discectomy.

**Figure 4:**
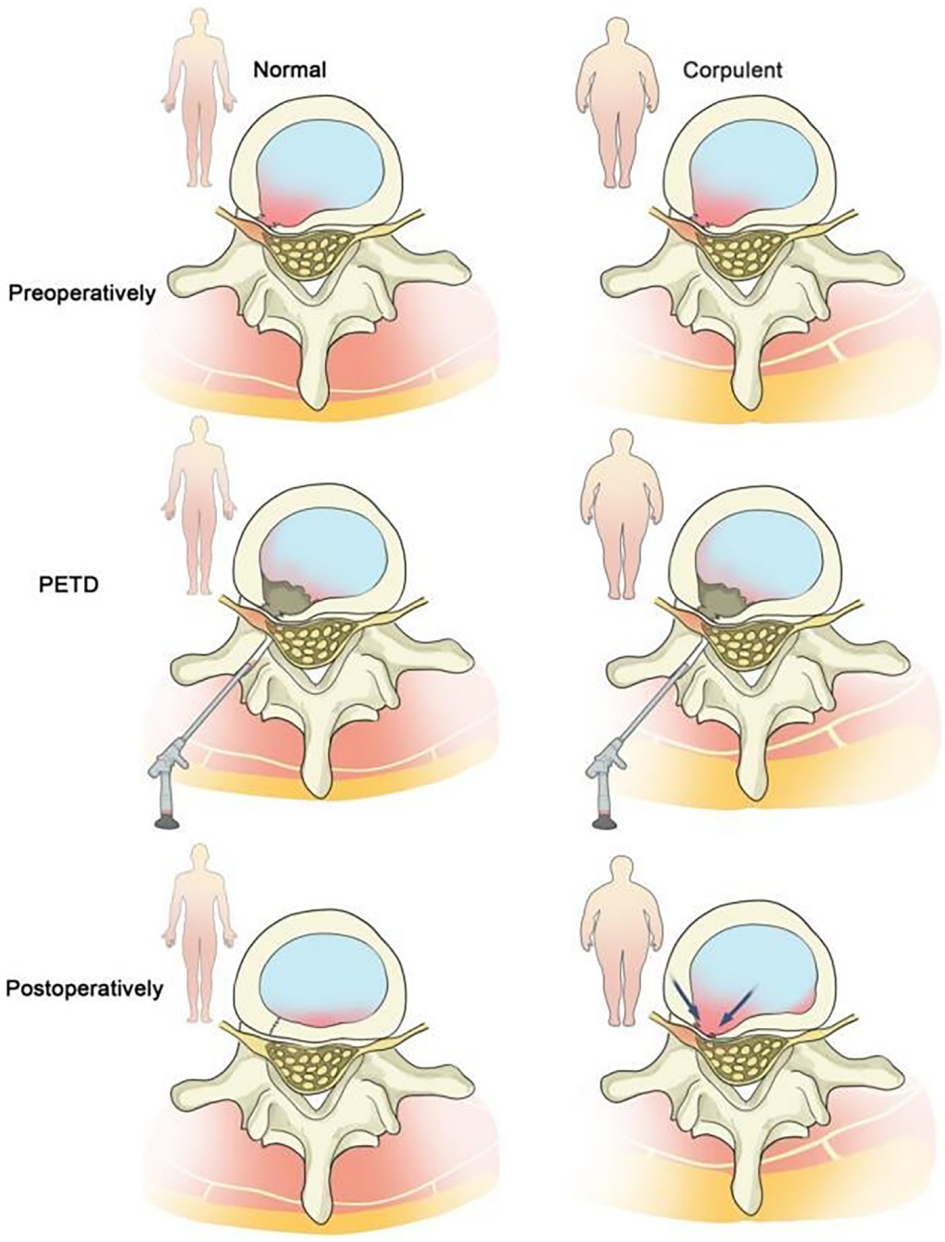
Obese patients are more prone to rLDH after PTED compared to patients with normal body weight.

Obesity, defined by a Body Mass Index (BMI) ≥ 30 kg/m² (43), is also widely recognized as a risk factor for recurrent lumbar disc herniation (rLDH) following various surgical interventions, including percutaneous transforaminal endoscopic discectomy (PTED) (18, 44, 45). Existing literature has confirmed that obesity is associated with reduced lumbar disc height (46, 47). The increased body weight in obese individuals exerts greater forces on spinal motion segments, particularly the lumbar spine, thereby delaying the healing of the annulus fibrosus through mechanical overload. In obese patients, increased flexion of the sacroiliac joints leads to degeneration of the joint surfaces. The altered biomechanical stability generates high torque, which can tear the annular structure (48–52). The disordered internal environment further affects the biosynthesis of extracellular matrix components in the outer region of the intervertebral disc (53–56). Therefore, after lumbar discectomy, the incision site of the annulus fibrosus in obese patients may become a weak point due to scar formation or non-healing, leading to reherniation of the nucleus pulposus. Based on these findings, we propose that the incidence of rLDH is higher in obese patients than in the general population (Figure 4). Thus, we selected obese patients as the subjects of our study, which is more in line with clinical needs.

The suture technique for a ruptured annulus was initially referenced in a sheep model study, where it was hypothesized that direct repair of annular incisions through tight suturing in sheep undergoing lumbar discectomy would not significantly affect the healing strength of the IVD (29). The advent of a simplified anular repair device heralds a new dawn for the practical clinical application of suturing the annular defects. Regrettably, in the study conducted in 2013, although suturig the annular defects does not adversely affect the positive patient outcomes inherent after a lumbar discectomy, the data are insufficient to conclusively demonstrate the effectiveness of suturing the annular defects in reducing the need for subsequent reoperations due to rLDH (28). At that juncture, the suturing technique is not yet fully developed, and the lack of a more simplified suturing device greatly increases the cost of repairing the annulus defects. This situation, where the benefits gained are not proportional to the costs paid, makes it uncertain whether the suturing of annular defects has achieved its anticipated benefits. In fact, due to the large surgical wound and extensive exposure range associated with conventional lumbar discectomy, there is significant destruction to the surrounding neural environment, often leading to extensive adhesions postoperatively. Under such conditions, the suturing technique is akin to a drop in the ocean. It is essential to emphasize that suturing the annular defects may merely enhance resistance to pressure through mechanical blocking, rather than by promoting healing. The healing of the annulus is a gradual process, not driven by direct repair. Significant damage to the normal anatomy largely overshadows the protective role of suturing in the gradual healing process. PTED is a way to remove protruding or trapped disc fragments without doing a full discectomy. It helps avoid the risks of spinal instability that come with doing a full discectomy in traditional LDH surgeries. The safety and efficacy of PTED have been continuously improved with the effective prevention of various complications arising from the ongoing refinement of PTED techniques (57–59). PTED exhibits comparability to traditional lumbar discectomy with respect to therapeutic efficacy and safety in the treatment of LDH, possessing evident minimally invasive benefits and facilitating rapid postoperative recovery. However, it doesn't show a statistically significant advantage over lumbar discectomy when it comes to the rate of recurrence and the number of cases of recurrent radicular pain after surgery. To address the issue of rLDH after PTED, there are currently only a few reports on the use of the Disposable Annulus Stapler to address annular defects following PTED. It remains uncertain whether the AFS can be widely and effectively applied in clinical practice to significantly reduce the incidence of rLDH. In a network meta-analysis conducted in 2021, researchers found that in the SUCRA plot for pain scores, percutaneous endoscopic discectomy and annulus fibrosus repair ranked second and third, respectively, just after open discectomy (60). This not only highlights the significant potential of PTED combined with AFS in reducing the recurrence of disc herniation after discectomy, but also provides inspiration for our research.

Previous studies have suggested that obesity is a significant factor for recurrence after PTED and can also affect the incidence of postoperative disc reherniation. Data from these research suggest that the long-term recurrence rate after PTED in obese patients can reach −5–10 percent within a one-year follow-up period (61–63). The proportion of obese patients who experience severe pain immediately postoperatively due to incomplete decompression of lumbar disc herniation on the nerve root, necessitating intervention with conventional microdiscectomy on the same postoperative day, can even reach up to 10% (63). The data from these references have provided prospective conditions for our research. In this retrospective study, our suturing technique differs from the conventional single-stitch method by incorporating the Disposable Annulus Stapler. Specifically, the loop of the second stitch is pre-threaded over the suture of the first stitch, enabling the first knot to be secured simply by tightening the suture. This ensures the stability of the suture knot. This approach reduces the prognostic variability associated with technical errors in traditional single-stitch suturing and significantly decreases the time required for physicians to learn the suturing technique. As a result, it ensures the reliability of annular fibrosus suturing within a shorter timeframe, thereby enhancing the reliability of the study outcomes. In our investigation, we observed that obese patients who underwent PTED combined with AFS did not require secondary traditional discectomy interventions within the short term, as previously reported in the literature. Additionally, no instances of recurrence were documented throughout the one-year follow-up duration. Individuals who received PTED in conjunction with AFS exhibited marked alleviation of postoperative pain and substantial enhancements in quality of life, with no instances of reherination. Our findings further substantiate the reliability of PTED combined with AFS in treating LDH and reducing the incidence of recurrent radicular leg pain following PTED. The synergy of AFS and PTED is fundamentally attributed to its ability to address the underlying cause of LDH. The procedure facilitates early closure of the annular defects, which prevents residual nucleus pulposus in obese patients from re-herniating through the annular defect under mechanical stress and reduces the release of inflammatory mediators within the disc that could irritate nerve roots. Therefore, closing the annular defects not only reduces the short-term recurrence of disc herniation after surgery but also effectively decreases the mechanical irritation of the ruptured and bulging annulus on the nerves, thereby alleviating the postoperative residual symptoms of low back pain and leg pain associated with recurrent disc herniation.

## 5 Limitations

The limitations of this study include the lack of a control group and insufficient sample size, which means that the accuracy and reliability of the results should be viewed with caution. Another limitation stems from the heterogeneity between this study and previous studies. Although the heterogeneity of the participant population itself is considered relatively low, such indirect comparisons may lead to further imprecision.

## 6 Conclusion

PTED combined with AFS alleviated patients' pain, improved their functional outcomes, and significantly reduced the likelihood of severe short-term complications and long-term recurrent leg pain during a 12-month observation period.

## Data Availability

The original contributions presented in the study are included in the article/Supplementary Material, further inquiries can be directed to the corresponding authors.
